# Microbial colonization and degradation of marine microplastics in the plastisphere: A review

**DOI:** 10.3389/fmicb.2023.1127308

**Published:** 2023-02-17

**Authors:** Xinyi Zhai, Xiao-Hua Zhang, Min Yu

**Affiliations:** ^1^Frontiers Science Center for Deep Ocean Multispheres and Earth System, College of Marine Life Sciences, Ocean University of China, Qingdao, China; ^2^Laboratory for Marine Ecology and Environmental Science, Laoshan Laboratory, Qingdao, China; ^3^Institute of Evolution and Marine Biodiversity, Ocean University of China, Qingdao, China

**Keywords:** microplastics, marine microorganisms, plastisphere, biodegradation, quorum sensing

## Abstract

Marine microplastic pollution is a growing problem for ecotoxicology that needs to be resolved. In particular, microplastics may be carriers of “dangerous hitchhikers,” pathogenic microorganisms, i.e., *Vibrio*. Microplastics are colonized by bacteria, fungi, viruses, archaea, algae and protozoans, resulting in the biofilm referred to as the “plastisphere.” The microbial community composition of the plastisphere differs significantly from those of surrounding environments. Early dominant pioneer communities of the plastisphere belong to primary producers, including diatoms, cyanobacteria, green algae and bacterial members of the *Gammaproteobacteria* and *Alphaproteobacteria*. With time, the plastisphere mature, and the diversity of microbial communities increases quickly to include more abundant Bacteroidetes and *Alphaproteobacteria* than natural biofilms. Factors driving the plastisphere composition include environmental conditions and polymers, with the former having a much larger influence on the microbial community composition than polymers. Microorganisms of the plastisphere may play key roles in degradation of plastic in the oceans. Up to now, many bacterial species, especially *Bacillus* and *Pseudomonas* as well as some polyethylene degrading biocatalysts, have been shown to be capable of degrading microplastics. However, more relevant enzymes and metabolisms need to be identified. Here, we elucidate the potential roles of quorum sensing on the plastic research for the first time. Quorum sensing may well become a new research area to understand the plastisphere and promote microplastics degradation in the ocean.

## 1. Introduction

Plastics have the advantages of low density, good ductility, durability and low cost, and have become widely used, worldwide. The current use of plastics is ~20 times that of half a century ago, and it is expected that the production and use will double in the next 20 years (Urbanek et al., 2018). Most of the plastic wastes discharged into the environment are chemically stable, corrosion-resistant, and difficult to be degraded by microorganisms (Lambert and Wagner, 2016; Yang et al., 2020). This has resulted in a severe environmental issue (Wright et al., 2020). It is predicted that 4.8–12.7 million tonnes of plastics enter the oceans annually. This amount is likely to increase by an order of magnitude within the next decade (Jambeck et al., 2015; Wright et al., 2020). Recently, the COVID-19 pandemic has driven explosive growth in the use of face masks resulting in many issues related to their disposal (Wang et al., 2021). Indeed, it is estimated that over 129 billion face masks are used globally each month, and over 16 million plastic particles are released from just one weathered mask by the effect of sand (Prata et al., 2020). Plastics could be classified by physical and chemical properties, such as density, molecular weight, crystallinity and functional groups as well as the additives used, such as plasticizers, antioxidants and flame retardants (Shah et al., 2008; Krueger et al., 2015). The main types of “nonbiodegradable” plastics include polyethylene (PE), polypropylene (PP), polystyrene (PS), polyethylene terephthalate (PET) and polyvinylchloride (PVC).

**Figure 1:**
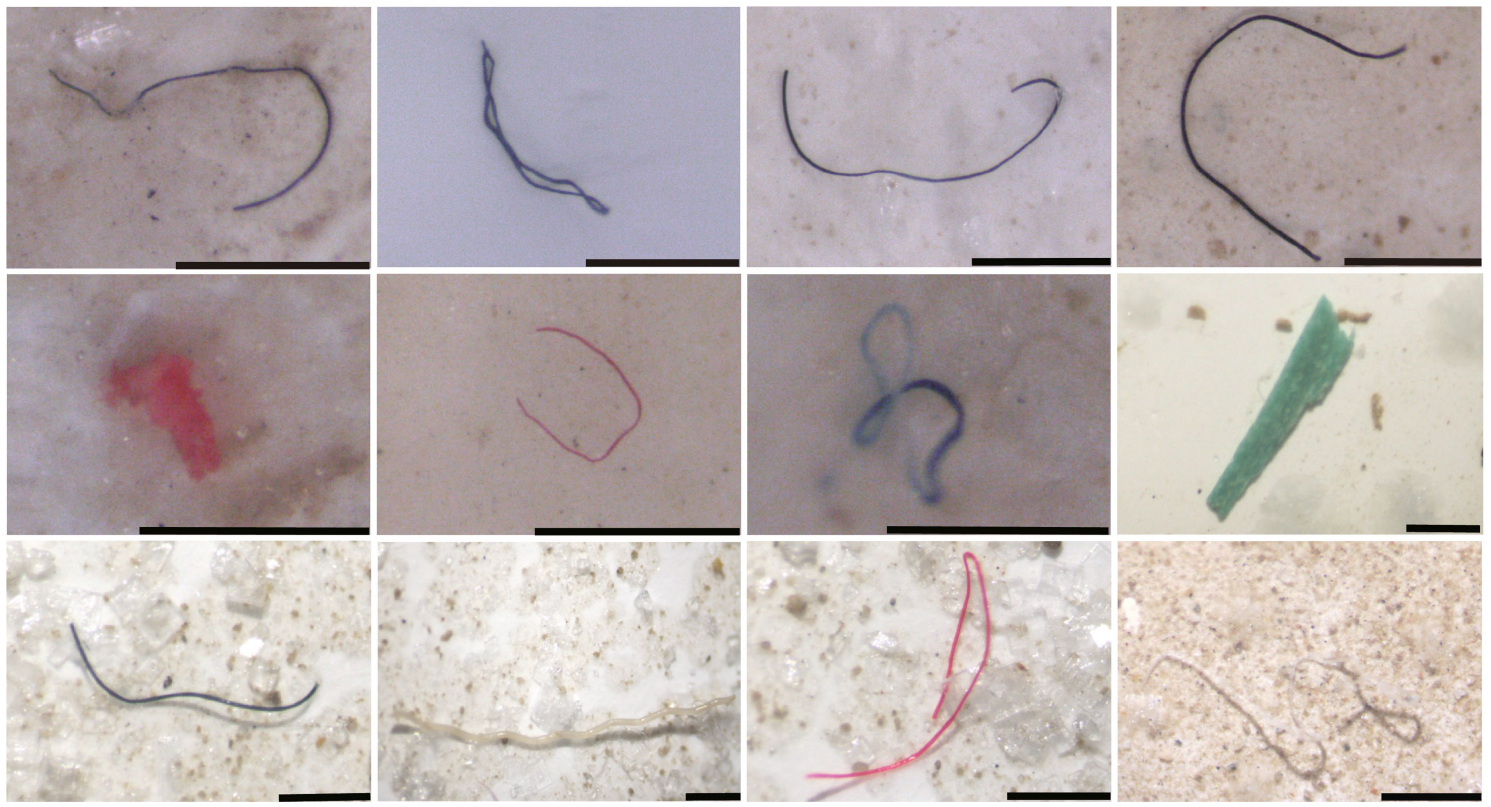
Microplastics in the coast of Qingdao, China. The black line refers to 500 μm.

Microplastics (MPs) refer to the plastic fragments of <5 mm in diameter, and may be divided into primary and secondary MPs. Primary MPs are directly produced and used in personal care products, such as toothpaste and certain cosmetics, and cause direct microplastic pollution after discarded to the environment (Zhang et al., 2018). Secondary MPs refer to the plastic fragments that are broken up by photodegradation, mechanical breakup, erosion and aquatic immersion (Akdogan and Guven, 2019; Cheng et al., 2020), which are main form of MPs present in the ocean. It is estimated that there are more than 5.25 × 10^12^ plastics in the surface waters of the world’s ocean, of which the weight of MPs is ~0.35 × 10^5^ tonnes (Eriksen et al., 2014). MPs could be transported horizontally for long distances through the ocean currents and wind, and vertically depending on the density of the particles. At present, MPs have been found to be widely distributed in the oceans, from polar regions (Peeken et al., 2018) to the equator, from densely populated areas to remote islands (Ivar do Sul et al., 2009), and from beaches (Claessens et al., 2011) to large ocean gyres and deep seas (Pichel et al., 2007; Van Cauwenberghe et al., 2013). In addition, MPs has been detected in the deepest trenches, i.e., the Mariana Trench (Peng et al., 2018). For example, the MPs isolated from the coast of Qingdao, China are detailed in Figure 1.

MPs have serious toxic effects on marine organisms and human beings, as well as negative effects on the ecological environment. Firstly, plasticizers with ecological toxic effects are released into the marine environment. Secondly, MPs can combine with persistent organic matter to form composite pollutants that cause enhanced toxicological effects (Ahmad et al., 2020). Thirdly, MPs’ surface structure may be changed, and become charged in the seawater thereby increasing toxicity that may be caused by interaction with heavy metals. The toxic substances may be accumulated in marine animals after they ingest MPs, which lead to damage to the biodiversity in marine environments (Ahmad et al., 2020). Moreover, MPs may be enriched by passage through the human body leading to adverse effects on health (Amobonye et al., 2021). Recently, increasing concern has been raised regarding the hazard potential of microplastic-associated microbial communities. In particular, certain MP surfaces have been shown to exhibit selective enrichment of bacterial pathogens—so called “dangerous hitchhikers”—that disperse over long distances (Kirstein et al., 2016; Rummel et al., 2017). For example, *Vibrio* species are enriched particularly on MPs (Zettler et al., 2013; Frère et al., 2018; Kesy et al., 2019). The ability of MPs to serve as carriers for dangerous hitchhikers poses threats to both aquatic ecosystems and human health. Besides colonization with pathogens, MPs could act as a long-term reservoir for antibiotic resistance (Liu et al., 2021).

The damage of MPs to biodiversity and ecosystem health is increasing in significance, especially with regards to microorganisms. Elucidating the role of MPs in the microbial loop and the role of microorganisms in the degradation of MPs is of great value (Amaral-Zettler et al., 2020). This may provide insights for the design of efficient degradation enzymes, and thereby reduce damage to human beings and the environment. This article reviews the current knowledge of the bacterial composition of the MP biofilms, the biodegradation of MP, and the relationship between plastisphere and quorum sensing (QS).

## 2. Formation of the plastisphere

MPs can provide advantages to the colonizing microbial communities, including physical support, nutrient supply and habitats that help microorganisms to resist harsh environmental conditions (Oberbeckmann and Labrenz, 2015; Shen et al., 2019; Ahmad et al., 2020). As with any kind of surface, microorganisms attach to and colonize MP surfaces immediately after the material enter the aquatic ecosystem, forming MP biofilms that have been termed the “plastisphere” (Zettler et al., 2013). The plastisphere may be regarded as a new micro-ecosystem in pelagic waters, and include primary producers, predators, symbionts, and decomposers (Amaral-Zettler et al., 2020). The microbial communities in the plastisphere are composed of a diverse range of bacteria, fungi, viruses, archaea, algae, and protozoans (Wright et al., 2020).

### 2.1. Formation and succession

**Figure 2:**
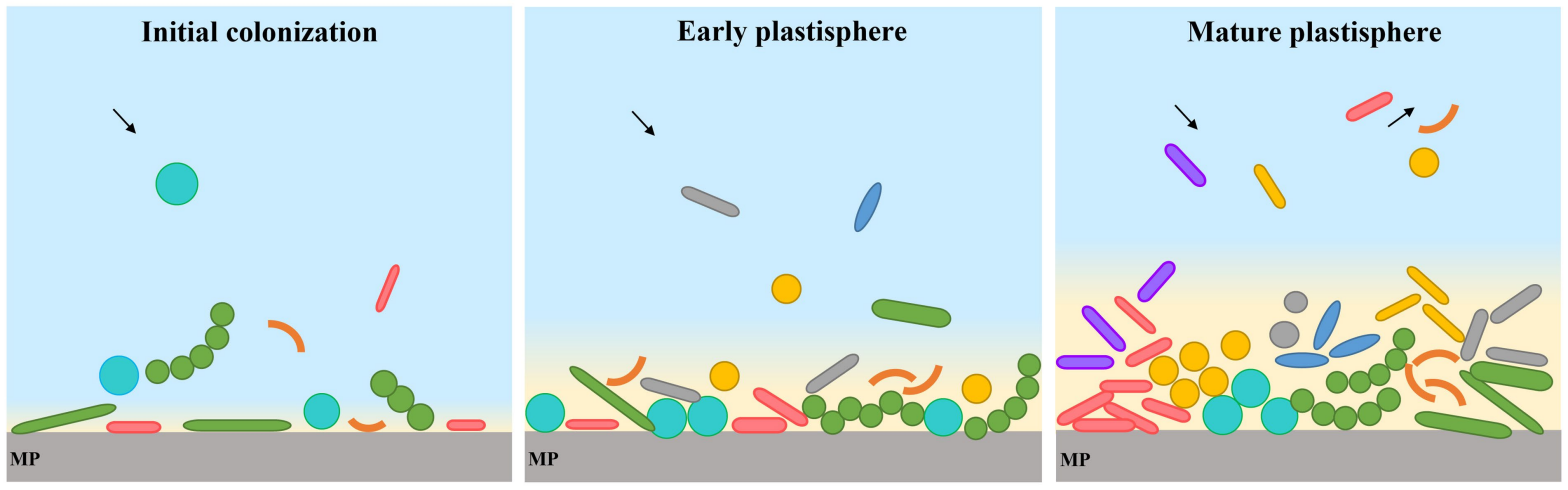
The formation of and succession in the plastisphere.

The growth of the microbial community on MP biofilms represents a temporal succession process that may be divided into different stages (Yang et al., 2020). In the initial colonization stage, the pioneer microorganisms are able to form microbial assemblages, and cover the maximum possible surface area of plastic. These generalist organisms proliferate without competition other than to grow faster than the rest to cover the surface (Wright et al., 2020), and pave the way for the enhanced attachment of other microorganisms, and recycling of resources (Onda and Sharief, 2021). As the biofilm grows, within days microorganisms attach to the plastic surface irreversibly, followed by a succession of other bacteria, viruses and eukaryotic microorganisms (Onda and Sharief, 2021). This is achieved by pili, adhesion of proteins, and production of complex extracellular polymeric substances (EPS), which facilitate adhesion to surfaces and stablish cell–cell interactions (Dussud et al., 2018). The secondary metabolites may improve quorum sensing and the antimicrobial effect to resist competing microorganisms (Davey and O’Toole, 2003; Hall-Stoodley et al., 2004; Carozza et al., 2015). Processes involve catalyzing metabolic reactions, including adsorption, desorption, and fragmentation of microplastic-associated compounds (Harrison et al., 2011; Urbanek et al., 2018). Finally, the continuous recruitment, loss, and replacement of species transform the structure into a hotspot for diverse interacting communities (Onda and Sharief, 2021), resulting in a mature plastisphere. For example, maturation phase of the PE plastisphere appeared after 30 days immerging in seawater, and a stabilization of bacterial abundance and heterotrophic bacterial production without significant increase was reached (Cheng et al., 2020). However, there was a drastic shift in bacterial community structure in maturation phase (Cheng et al., 2020). The formation of and succession in the plastisphere is described in Figure 2.

Early dominant pioneer communities in marine and estuarine plastispheres belong to primary producers including diatoms, cyanobacteria, green algae (Zettler et al., 2013; Bryant et al., 2016) and bacterial members of *Gammaproteobacteria* and *Alphaproteobacteria* (Lee et al., 2008; Oberbeckmann and Labrenz, 2015). As for *Gammaproteobacteria*, *Oceanospirillaceae* and *Alteromonadaceae* representatives were present, and which could occur up to 59.1% of the total microbial community after 1 day of immersion of the MPs in the marine habitat. Their presence decreased significantly later (Pollet et al., 2018; Roager and Sonnenschein, 2019). Also, *Vibrio* spp. were early colonizers of PE and PS in marine environments (Kesy et al., 2019). *Alcanivorax*, *Aestuariicella*, and *Marinobacter* were the dominant early colonizers on different plastic substrates (Dussud et al., 2018). In addition, the families *Saprospiraceae* and *Oscillatoriaceae* were found on early stages of the plastisphere (Oberbeckmann et al., 2016). With the plastisphere becoming mature, the abundance of distinct microbial communities, including Bacteroidetes (Lee et al., 2008) and *Alphaproteobacteria* (Pollet et al., 2018), increased quickly on the plastisphere. For example, Bacteroidetes were always significantly more abundant at later stages (Hadad et al., 2005). In contrast, *Rhodobacteraceae* came early to MPs, and they were persistent and became dominant as time passed (Pollet et al., 2018). *Rhodospirillaceae,* including purple sulfur bacteria, may fix N_2_ and thus provide “fertilizer” to other community members. In addition, flavobacteria, *Planctomycetaceae* and *Phyllobacteriaceae* were abundant at the later stages of the MPs colonization (Pinto et al., 2019).

### 2.2. Microbial community composition

The microbial community composition of the plastisphere represents the main research target, and many studies revealed significant differences from microhabitats in the same environment (Bryant et al., 2016; Oberbeckmann et al., 2018; Ogonowski et al., 2018). Proteobacteria, Bacteroidetes, Firmicutes and Cyanobacteria are usually the main bacteria enriched in the plastisphere. This differs from a range of other substrates, such as metal, rubber, glass, fabric, and seawater (Ogonowski et al., 2018; Woodall et al., 2018). Moreover, there was a different abundance and distribution pattern of photosynthetic and heterotrophic bacteria in plastic samples compared with those in seawater (Zettler et al., 2013). These data indicate that the plastisphere is a specific ecological niche where microorganisms with potential metabolic adaptations, specific metabolic pathways (including attachment, chemotaxis, additive resistance, degradation, secretion, nitrogen fixation and motility) were enriched (Roager and Sonnenschein, 2019). Studies on microbial community composition of the plastisphere based on research methods of field sampling and *in situ* incubation are summarized in Table 1.

**Table 1:** Studies on the plastisphere based on sampling site and time, plastic type and microbial community.

Sampling site	Month	Plastic types	Core communities	Research method	References
Western Mediterranean Sea	4–11	PE PP PS	Cyanobacteria (40.8%, mainly *Pleurocapsa* sp. *Oscillatoriales*, *Chroococcales*, *Nostocales* and *Pleurocapsales* orders, *Synechococcus* sp., *Calothrix* sp., *Scytonema* sp. and *Pleurocapsa* sp.) *Alphaproteobacteria* (32.2%, mainly *Roseobacter* sp.)	Field sampling	Dussud et al. (2018)
The North Atlantic	10, 12	PE PP PS	*Alphaproteobacteria* (17.67% ± 5.28%) *Gammaproteobacteria* (40.76% ± 8.43%) *Flavobacteria* (16.83% ± 2.64%) Cyanobacteria (9.09% ± 7.40%)	Field sampling	Frère et al. (2018)
The North Atlantic	6	PE PET PS	*Alphaproteobacteria*, *Streptomycetales*, Bacteroidetes and Cyanobacteria	Field sampling	Debroas et al. (2017)
North Sea	3, 6, 9	PE PP	*Alphaproteobacteria*, *Gammaproteobacteria* and Bacteroidetes	Field sampling	De Tender et al. (2015)
North Sea	9–7	PE	Proteobacteria and Bacteroidetes	*In situ* incubation	De Tender et al. (2017)
Mediterranean Sea	11	PE PP	Bacteroidetes, *Gammaproteobacteria* and *Alphaproteobacteria*	Field sampling	Delacuvellerie et al. (2019)
The Yangtze estuary	4	PE PP PS	Proteobacteria, Cyanobacteria, Bacteroidetes and *Actinobacteria*	Field sampling	Jiang et al. (2018)
The North Atlantic	5, 6, 7	PP PE	*Vibrio* sp.	Field sampling	Zettler et al. (2013)
The equatorial Atlantic	10–12	PVC PE PA	Proteobacteria (52%), Bacteroidetes (10%), and Crenarchaeota	Field sampling	Woodall et al. (2018)
The Eastern North Pacific	7	PE PP PS	*Bacillus bacteria* and *pennate diatoms*	Field sampling	Carson et al. (2013)
North Sea	1, 7	PE PP	Bacteriodetes, Cyanobacteria and Proteobacteria	Field sampling	Oberbeckmann et al. (2014)
Baltic Sea	8, 9	HDPE PS	*Flavobacteria*ceae, *Rhodobacteraceae* and *Planctomycetaceae*	*In situ* incubation	Oberbeckmann et al. (2018)
The North Pacific	8	PE PP	Cyanobacteria and *Alphaproteobacteria*	Field sampling	Bryant et al. (2016)
Baltic Sea	8	PE PP PS	*Alphaproteobacteria* and *Sphingomonadaceae*	*In situ* incubation	Ogonowski et al. (2018)
Mediterranean Sea	–	PVC	*Gammaproteobacteria* (*Oceanospirillaceae* and *Alteromonadaceae*), *Alphaproteobacteria* (*Rhodobacteraceae*) and Bacteroidetes (*Flavobacteriia*)	*In situ* incubation	Pollet et al. (2018)
Mediterranean Sea	–	PS	Proteobacteria and Firmicutes	Field sampling	Syranidou et al. (2019)
South China Sea	5, 8	PS	*Rhodobacteraceae* and *Sphingomonadaceae*	*In situ* incubation	Lee et al. (2014)
South China Sea, the Yellow Sea	4, 7, 11, 1	PP PVC	*Alphaproteobacteria* (*Rhodobacteraceae*) and *Gammaproteobacteria*	Field sampling	Xu et al. (2019)
Mediterranean Sea	8	PE	*Roseobacter*, *Oleiphilus*, and *Aestuariibacter*	*In situ* incubation	Erni-Cassola et al. (2020)
Baltic Sea	2	PE PP PS	*Gammaproteobacteria* (47%–81%), *Alphaproteobacteria* (7%–34%) and *Bacteroidia* (3%–20%)	*In situ* incubation	Hansen et al. (2021)
Mediterranean Sea	10	PE	*Gammaproteobacteria*, *Alphaproteobacteria* and Bacteroidetes	Field sampling	Sucato et al. (2021)
The Yellow Sea	8	–	*Vibrio*, *Pseudoalteromonas* and *Alteromonas*	*In situ* incubation	Sun et al. (2020)
North Sea	–	PE PP PS PVC PET	*Alphaproteobacteria* (18%–53%) and *Gammaproteobacteria* (20%–75%)	*In situ* incubation	Kirstein et al. (2019)
Southern Ocean	2 (summer in Southern Hemisphere)	HDPE	*Gammaproteobacteria* and *Betaproteobacteria*	Field sampling	Cappello et al. (2021)
Mangrove in China	4, 7	PP PS	*Vibrio*, *Rhodobacteraceae*, *Alteromonadaceae* and *Pseudoalteromonadaceae*	*In situ* incubation	Xie et al. (2021)
Bohai Sea	7, 8, 9	PVC PP PE PS PU	*Gammaproteobacteria* (*Vibrio*, *Pseudoalteromonas*) and Bacteroidetes	*In situ* incubation	Zhang et al. (2021b)

PE, polyethylene; PP, polypropylene; PS, polystyrene; PET, polyethylene terephthalate; PVC, polyvinylchloride; PU, polyurethane; HDPE, high density polyethylene.

#### 2.2.1. Diatoms

Diatoms (phototrophic organisms) are common residents of the plastisphere, and are often reported as early and even dominant colonizers on plastic debris (notably the plastics exposed to sunlight; Table 1). For example, diatoms dominated in the microbial community on the abundant MPs in the Eastern North Pacific Gyre (Carson et al., 2013). Although the dominate role of diatoms declines with plastisphere maturing, they remain consistent members in the plastisphere (Amaral-Zettler et al., 2020).

#### 2.2.2. Cyanobacteria

As another photosynthetic organism, Cyanobacteria join with diatoms to typically contribute net primary production on MPs, providing nutrients for heterotrophs in the plastisphere. Many studies have shown that Cyanobacteria are dominate candidates of the plastisphere, especially in the early stages (Table 1). For example, the genera *Stanieria* and *Pseudomorphium* were found abundant on PET across stations (Orr et al., 2004). In particular, the genus *Phormidium* was found commonly in microbial mat on various substrates, and has been found to be present on PE, PET, and PP samples (Table 1). This genus was identified as an important member of a plastic-colonizing community in the North Atlantic (Zettler et al., 2013), and also determined to degrade hydrocarbons (Antić et al., 2006). In addition, filamentous cyanobacteria, *Phormidium,* occurred on plastics, but was absent from seawater samples (Zettler et al., 2013). Sunlight exposure on floating MPs may well be beneficial to the enrichment of this photosynthetic organism on plastic debris.

#### 2.2.3. Proteobacteria

As the most abundant phylum globally, Proteobacteria comprise the most enriched phylum on the plastisphere in comparison with populations in seawater. As is shown in Table 1, within the phylum Proteobacteria, the classes *Alphaproteobacteria*, *Gammaproteobacteria*, *Betaproteobacteria*, and *Deltaproteobacteria* are enriched on plastic samples.

Many studies showed that the family *Rhodobacteraceae,* within class *Alphaproteobacteria,* occupied the core population of the plastisphere (Table 1). *Rhodobacteraceae*, especially *Roseobacter*, was recognized as a primary colonizer of various substrates in marine environments (Dang et al., 2008). However, these organisms were found to be enriched significantly on marine plastic debris as compared with organic particles (Dussud et al., 2018) and seawater (Erni-Cassola et al., 2020). In fact, *Rhodobacteraceae* is not only the primary colonizer, but is also abundant at all colonization periods, and exerts an important role in biofilm formation. This reflects the extracellular polymeric substance (EPS) produced by *Rhodobacteraceae* after colonizing new surfaces, leading to enhancing the settlement of other microorganisms (Amaral-Zettler et al., 2020). Apart from *Rhodobacteraceae*, bacteria belonging to the family *Erythrobacteraceae* have been found on various macro−/micro-plastics (Table 1). Moreover, hydrocarbonoclastic bacteria were overrepresented in the plastic fraction (Dussud et al., 2018).

Within *Gammaproteobacteria*, the order *Pseudomonadales* has been highlighted (Oberbeckmann et al., 2018; Sun et al., 2020; Zhang et al., 2021b). *Pseudomonas* was found to have biodegradation potential on MPs (as discussed later) leading to interest for MP-degradation research. As is shown in Table 1, *Vibrio* is another member of the *Gammaproteobacteria* detected widely on MPs in marine environments (Zettler et al., 2013; Sun et al., 2020; Xie et al., 2021). Many *Vibrio* spp. are pathogenic and regarded as dangerous hitchhikers on MPs facilitating their spread widely throughout the marine environment. *Vibrio* spp. are usually primary colonizers, and grow rapidly with the ability to dominate on the plastisphere in comparison with natural seston (Frère et al., 2018; Kesy et al., 2019). Moreover, the order *Oceanospirillales* and the family *Alcanivoraceae* have been found in the plastisphere (Oberbeckmann et al., 2016; Syranidou et al., 2019; Table 1).

#### 2.2.4. Bacteriodetes

Bacteriodetes is the second most represented bacterial phylum on plastic surfaces (Table 1). Here, the distribution of Bacteroidetes do not reveal clear temporal patterns, and is dominated constantly by *Flavobacteria.* These may well be essential members, and key players in the formation of biofilms (Pollet et al., 2018). The class *Flavobacteria* has been recognized as colonizers on various MPs, including PS, PE, PP, and PET (Table 1). For example, the family *Flavobacteriaceae* occurred more frequently on PET particles compared with glass beads (Oberbeckmann et al., 2016), and PE and PS compared with wooden pellets (Oberbeckmann et al., 2018). *Flavobacteriaceae* has been described as a keystone taxon in biofilms that feed off exudates from dinoflagellates and diatoms (Amin et al., 2012).

The order *Sphingobacteriales* and genus *Lewinella* have been reported frequently as colonizers of the plastisphere (Zettler et al., 2013; Oberbeckmann et al., 2016; Debroas et al., 2017; Jiang et al., 2018; Ogonowski et al., 2018). *Sphingobacteriales* was shown capable of metabolizing polycyclic aromatic hydrocarbons (PAHs; Leys et al., 2004) and abundant in both short-term (60 days) and long-term (5 months) MP samples after outside incubation in North Seawater (Kirstein et al., 2019).

#### 2.2.5. Firmicutes

Firmicutes was found among the most abundant populations enriched on marine PS samples (Syranidou et al., 2019). In particular, the genus *Bacillus* has revealed a wide-spread and powerful biodegradation potential on various kinds of MPs, namely PE, PP, PS, PVC, and PET.

### 2.3. Factors influencing the formation of the plastisphere

Factors driving the microbial composition in the plastisphere are complex, and may be divided into environmental and polymer characteristic factors. Environmental aspects include physicochemical factors (namely temperature, salinity, depth, pH, dissolved oxygen and light), nutrient availability (e.g., organic/inorganic carbon, ammonium, nitrate, nitrite and phosphorus) for microbial growth, pollutants (e.g., toxic metals, antibiotics and persistent organic pollutants), and biotic factors (principally algae and animals; Yang et al., 2020). Also, polymer characteristic factors include the type, surface property and size of the plastics.

Environmental factors related to geography have a larger influence on the plastisphere community than the plastic type (Oberbeckmann et al., 2018; Kesy et al., 2019). Studies on microbial communities on different polymer types floating in the North Pacific and North Atlantic demonstrated that plastisphere communities were well-defined biogeographically, displaying latitudinal gradients in species richness, and to a lesser extent between polymer types (Amaral-Zettler et al., 2016). As is shown in Table 1, core microbial communities in marine environments show clear geographical distribution patterns, which may influence the formation of specific plastispheres. So far, studies investigating the plastisphere in various marine environments are mainly focused in Europe (Mediterranean Sea, North Sea and Baltic Sea) and China, and to a lesser extent the Atlantic, Pacific and Southern Ocean (Table 1).

Apart from geography, there are other environmental factors influencing the plastisphere community. Microbial diversity and function show strong dependency on environmental conditions, in turn influencing MP biofilm formation and structure (Yang et al., 2020). And the early attachment and succession of plastisphere are affected by the growth rate of the biofilm, determined by factors including temperature, light, and nutrient availability (Wright et al., 2020). For instance, the light availability was shown to play a key role in shaping plastisphere communities, and revealed that environmental variables, not plastic type, had the largest impact on microbial composition (Wright et al., 2021). In addition, microbial communities in the plastisphere along an environmental gradient were shaped more by salinity than by plastic type (Oberbeckmann et al., 2018; Jacquin et al., 2019). Indeed, salinity gradients influenced microbial communities in the plastisphere significantly (Oberbeckmann et al., 2018), particularly enhancing the abundance of *Gammaproteobacteria* (Oberbeckmann et al., 2018; Kesy et al., 2019). Moreover, ocean acidification altered bacterial communities on marine plastic debris (Harvey et al., 2020). It was noteworthy that the light intensity was negatively correlated with depth, which could cause the depth-dependent distribution of photosynthetic bacteria. Photoautotrophic bacteria, such as cyanobacteria, i.e., the genera *Phormidium* and *Rivularia*, dominated the plastisphere communities living in surface seawater, whereas the core microbiome of the plastisphere living on the seafloor and sub-surface seawater appeared to comprise Bacteroidetes (*Flavobacteriaceae*) and Proteobacteria (*Rhodobacteraceae* and *Alcanivoracaceae*; Jacquin et al., 2019).

In addition, the abundance of the microbial community of the plastisphere is influenced by the size and especially the type of plastic (Frère et al., 2018; Wright et al., 2021). This may be attributed to the chemotactic selectivity colonization on MPs with different polymer chemical properties (Xie et al., 2021). The results in *a situ* exposure experiments for nine different types of MPs, including low density polyethylene (LDPE), PS, expanded polystyrene (EPS), PP, polycarbonate (PC), polyamide 6 (PA6), PVC, PET, and acrylonitrile butadiene styrene (ABS) in mangrove, revealed that MPs with different chemical structures would attract different microbial colonizers (Xie et al., 2021). Hansen et al. (2021) quantified the biofilm on PE, PP, and PS by six marine bacterial strains (*Vibrio*, *Pseudoalteromonas* and *Phaeobacter*), demonstrating that each one had a unique colonization behavior with either a preference for PS or PP over the other polymer types, or no preference at all. Wright et al. (2021) re-analyzed all plastisphere studies that utilize 16S rRNA gene amplicon sequencing, and found that *Oceanospirillales* and *Alteromonadales* were more abundant in plastic samples than control biofilms, with *Oceanospirillales* generally being more abundant in aliphatic plastic samples, and *Alteromonadales* in other plastics. In addition, the roughness, hydrophobicity and age of MPs could clearly influence microbial community structure of plastisphere (Yang et al., 2020). Interestingly, differences in microbial composition between polymers are usually caused by rare taxa whereas the abundant microbial groups remain largely unchanged (Ivk et al., 2018).

Plastisphere development does rely largely on the surrounding microbial communities although there are significant differences between them (Arias-Andres et al., 2018). In fact, consistent conclusions have not been made when studying the microbial community compositions of the plastisphere in marine environments because of the variety of plastic substrate types, season and geographical location (Oberbeckmann et al., 2014). For example, early attachment and succession of the plastisphere depend on the growth rate of the biofilm, determined by physicochemical factors and nutrient availability. There may well be some polymer-specific communities in the initial colonization, which are largely influenced by the differences between materials, such as additives or preweathering, and will eventually converge over time (Amaral-Zettler et al., 2020).

### 2.4. Ecological effects of the plastisphere

The plastisphere has multiple effects on the fate of plastics and the marine environment (Cheng et al., 2020). Firstly, the plastisphere may change the density of MP particles, making them susceptible to vertical transport by sinking and floating (Lagarde et al., 2016). Secondly, the plastisphere may change the adsorption state of the MP surface, and affect the decomposition rate of the MPs in seawater. In the biofilm, autotrophic microorganisms may accelerate the oxidative decomposition of MPs by producing oxygen during photosynthesis. Heterotrophic microorganisms may degrade MPs by a series of processes, including biodeterioration, bio-fragmentation, bio-assimilation and biomineralization. Conversely, the plastisphere may slow down the photochemical decomposition process of MPs by ultraviolet light (Onda and Sharief, 2021). Moreover, microorganisms colonizing MPs may be transported vertically and for long-distances horizontally in the ocean, which may promote the diffusion of invasive species to fragile ecosystems (Gregory, 2009). Pathogens, such as *Vibrio*, *Tenacibaculum*, *Pirellulaceae* (Wright et al., 2021) and spirochetes (Xie et al., 2021), were usually found on MPs. When algal blooms and infectious diseases caused by pathogens occur, the negative influence on marine ecosystems is significant (Zettler et al., 2013; Rummel et al., 2017). Therefore, the risk of MPs carrying pathogens to infect humans and cause long-distance transmission should be determined (Xie et al., 2021).

## 3. Biodegradation of microplastic

The biodegradation of plastics may be defined as the process that microorganisms digest and decompose plastic fragments into available carbon sources by changing the plastics functional group structure, molecular weight, tensile property and other features associated with the polymers. The microbial degradation of MPs is affected by microorganisms, MPs properties, and environmental factors.

### 3.1. Microorganisms involved in microplastic/plastic degradation

Up to now, information regarding the microorganisms involved in plastic degradation is rudimentary. Currently, the microorganisms capable of degrading MPs are mainly associated with the terrestrial environment, and more so for PE degradation, and include *Achromobacter*, *Acinetobacter*, *Arthrobacter*, *Aspergillus* (fungus), *Bacillus*, *Comamonas*, *Delftia*, *Micrococcus*, *Nesiotobacter*, *Paenibacillus*, *Pseudomonas*, *Rahnella*, *Staphylococcus*, and *Stenotrophomonas*. These have been isolated from various soil environments, saltpan water and landfills (Table 2). Bacteria capable of degrading PET include *Ideonella* and *Saccharomonospora,* which were isolated from a PET bottle recycling site and recovered from compost, respectively (Kawai et al., 2014). *Pseudomonas*, *Bacillus* and *Achromobacter* were demonstrated to degrade PVC (Das et al., 2012; Giacomucci et al., 2019). *Aspergillus* showed the ability to degrade PU (Khan et al., 2017). Moreover, *Thermus*, *Bacillus* and *Geobacillus,* which were isolated from a sludge composting pool, degraded PS, suggesting that hyperthermophilic composting was a promising strategy for MPs removal from organic wastes (Chen et al., 2020). *Bacillus* and *Pseudomonas,* capable of degrading high impact polystyrene (HIPS), were obtained from landfills (Mohan et al., 2016). *Aspergillus*, *Bacillus*, *Pseudomonas*, *Staphylococcus*, and *Streptococcus* were isolated from different soil samples, and were capable of degrading PET and PS (Asmita et al., 2015). In addition, a *Rhodococcus* strain isolated from soil burying PE mulch was capable of degrading both PS and PE (Orr et al., 2004; Mor and Sivan, 2008).

**Table 2:** Bacteria capable of degrading MPs.

Plastic types	Microorganism	Environment	Sampling site	Research progress	Reference
LDPE	*Bacillus mycoides Acinetobacter baumannii Pseudomonas fluorescens Staphylococcus cohnii Staphylococcus xylosus*	Land	Waste coal soil in Poland	Isolation and culture	Nowak et al. (2011)
LDPE	*Bacillus cereus Micrococcus luteus Micrococcus lylae Rahnella aquatilis Arthrobacter viscosus*	Land	Forest soil in Poland	Isolation and culture	Nowak et al. (2011)
LDPE	*Bacillus pumilus Paenibacillus macerans Bacillus thuringiensis Bacillus amyloliquefaciens Micrococcus luteus*	Land	Crater soil in Poland	Isolation and culture	Nowak et al. (2011)
LDPE	*Bacillus Paenibacillus*	Land	Landfill site in Korea	Isolation and culture	Park and Kim (2019)
LDPE	Aspergillus clavatus^*^	Land	Landfill soil in India	Isolation and culture	Gajendiran et al. (2016)
LDPE	*Rhodococcus ruber*	Land	Soil burying PE mulch in Israel	Isolation and culture	Oberbeckmann et al. (2014)
LDPE	*Nesiotobacter exalbescens Bacillus vietnamensis*	Land	Saltpan water in India	Isolation and culture	Deepika and Madhuri (2015)
LDPE	*Pseudomonas*	Land	Waste dumping ground soil in India	Hydrolase	Tribedi and Sil (2014)
LDPE	*Bacillus Enterobacter asburiae*	Worm gut	Waxworm gut	Isolation and culture	Yang et al. (2014)
LDPE	*Pseudomonas aeruginosa*	Marine	Soil from an oil-spill beach in Korea	Alkane monooxygenase, rubredoxin, rubredoxin reductase	Jeon and Kim (2015)
LDPE	*Kocuria palustris Bacillus pumilus Bacillus subtilis*	Marine	Sea water of the Arabian Sea coast in India	Isolation and culture	Harshvardhan and Jha (2013)
LDPE HDPE	*Bacillus sphericus Bacillus cereus*	Marine	Shallow waters of the Indian Ocean	Isolation and culture	Sudhakar et al. (2007)
HDPE	*Delftia Comamonas Stenotrophomonas*	Land	Plastic debris from Cerrado Soil in Brazil	Isolation and culture	Peixoto et al. (2017)
HDPE	*Achromobacter xylosoxidans*	Land	Polyethylene bags and soil of waste sites in Poland	Isolation and culture	Kowalczyk et al. (2016)
HDPE	*Arthrobacter Pseudomonas*	Marine	Soil of coastal plastic waste Dumped sites in India	Isolation and culture	Balasubramanian et al. (2010)
HDPE	Aspergillus tubingensis^*^ Aspergillus flavus^*^	Marine	Plastic waste dumped Marine environmental site in India	Isolation and culture	Sangeetha Devi et al. (2015)
HDPE	*Pseudomonas Lysinibacillus*	Marine	Deep-sea sediment from Pelotas Basin in Brazil	Isolation and culture	Oliveira et al. (2021)
PE	Zalerion maritimum^*^	Marine	Strain culture	Laccase	Paço et al. (2017)
PE PVC	*Bacillus*	Marine	Sea water of Gujarat coast in India	Isolation and culture	Kumari et al. (2019)
PE PET	*Exiguobacterium*, *Halomonas*, *Ochrobactrum* sp	Marine	Coast of Qingao, China	Esterase and hydrolase	Gao and Sun (2021)
PET	*Alcanivorax Hyphomonas Cycloclasticus*	Marine	Messina Strait	Isolation and culture	Denaro et al. (2020)
PET	*Stanieria Pseudomorphium*	Marine	The North Sea off the United Kingdom coast	KEGG	Orr et al. (2004)
PET	*Ideonella sakaiensis*	Land	Sediment from PET bottle recycling site in Japan	PETase MHETase	Yoshida et al. (2016)
PET	*Saccharomonospora viridis*	Land	Compost in Japan	Cutinase	Kawai et al. (2014)
PET PS	Pseudomonas aeruginosa Bacillus subtilis Staphylococcus aureus Streptococcus pyogenes Aspergillus niger^*^	Land	Five different soil samples in India	Isolation and culture	Asmita et al. (2015)
PS	*Rhodococcus ruber*	Land	Soil burying PE mulch in Israel	Isolation and culture	Mor and Sivan (2008)
PS	*Thermus Bacillus Geobacillus*	Land	Sludge composting plant in China	Isolation and culture	Chen et al. (2020)
PS	Bacillus anthracis Enterobacter hormaechei Aspergillus niger^*^	Worm gut	Mealworm gut	Isolation and culture	Kong et al. (2018)
PS	*Exiguobacterium*	Worm gut	Mealworm gut	Isolation and culture	Yang et al. (2015a,b)
PS PE PET PP	*Bacillus.cereus Bacillus.gottheilii*	Mangrove	Mangrove sediment in Malaysia	Isolation and culture	Auta et al. (2017)
HIPS	*Bacillus Pseudomonas*	Land	Plastic dump yard soil in India	Esterase	Mohan et al. (2016)
PP	*Bacillus Rhodococcus*	Mangrove	Mangrove sediment in Malaysia	Isolation and culture	Auta et al. (2018)
PVC	*Pseudomonas citronellolis Bacillus flexus*	Land	Strain culture	Pure culture	Giacomucci et al. (2019)
PVC	*Pseudomonas aeruginosa Achromobacter*	Land	Crude oil contaminated soil in India	Isolation and culture	Das et al. (2012)
VC	*Ochrobactrum Pseudomonas putida*	Land	Waste site groundwater in America	Alkene monooxygenase	Danko et al. (2004)
PU	Aspergillus tubingensis^*^	Land	Dumping area in Pakistan	Isolation and culture	Khan et al. (2017)

* Fungus.

PE, polyethylene; HDPE, high density polyethylene; LDPE, low density polyethylene; PP, polypropylene; PS, polystyrene; PET, polyethylene terephthalate; PVC, polyvinylchloride; PU, polyurethane; VC, vinyl chloride.

It is noteworthy that *Bacillus* and *Enterobacter* in waxworm gut could “eat” PE (Yang et al., 2014), whereas *Exiguobacterium* (Yang et al., 2015a,b), *Bacillus anthracis*, *Enterobacter hormaechei*, and *Aspergillus niger* (Kong et al., 2018) in mealworm gut could attack PS. In addition, the marine polychaete *Marphysa sanguinea* inhabiting PS debris was able to degrade PS particles (Jang et al., 2018). Certainly, studying the microfauna and gut microorganisms may be an innovative way for the treatment of plastic waste.

At present, studies on the microbial degradation of marine plastics are limited, and mainly involve the enrichment and isolation of plastic degrading microorganisms in the offshore environment. Thus, *Arthrobacter*, *Aspergillus*, *Bacillus*, *Kocuria*, *Pseudomonas* and *Zalerion maritimum* (fungus) demonstrated the capability of degrading PE, and *Bacillus* could degrade PVC (Table 2). *Pseudomonas,* isolated from plastics immersed for several days in Bay of Bengal, was found have a great ability to degrade PP, LDPE and HDPE (Sudhakar et al., 2007). Furthermore, *Pseudomonas aeruginosa* E7, which was isolated from beach soil contaminated by an oil spill, displayed high degradative activity to low-molecular-weight polyethylene (LMWPE), and metabolized 40.8% of the carbons of LMWPE into CO_2_ after 80 days of composting (Jeon and Kim, 2015). *Pseudomonas* and *Lysinibacillus,* which were isolated from deep-sea sediment, used an increased extracellular matrix to improve cell adhesion to weathered HDPE (Oliveira et al., 2021). Other examples include *Rhodococcus* and *Bacillus,* which were isolated from mangrove, and were capable of degrading several kinds of MPs (Auta et al., 2017, 2018). Moreover, after 40 days of incubation, weight losses of PE, PET, PP, and PS particles were found and it was resulted from the action of *Bacillus cereus* and *Bacillus gottheilii* (Auta et al., 2017). *Bacillus* sp. strain 27 and *Rhodococcus* sp. strain 36 could also degrade PP and cause weight losses (Auta et al., 2018). Apart from aerobic bacteria, anaerobic marine consortia were found degrade PVC (Giacomucci et al., 2020).

As is shown in Table 2, *Bacillus* and *Pseudomonas* were very important bacteria in the degradation of MPs. Among the 15 strains of bacteria capable of degrading HDPE isolated from the coast, *Pseudomonas* was the most efficacious (Balasubramanian et al., 2010). *Pseudomonas* spp. achieved a percentage weight reduction of PE of up to 20% within 120 days (Kyaw et al., 2012). The metabolites of PS decomposed by *Pseudomonas* were styrene, toluene and benzene, which could be easily used by other bacteria (Shah et al., 2008). A novel carboxylic ester hydrolase was identified in the genome of the marine bacterium *Pseudomonas aestusnigri*, indicating its significant potential for PET degradation (Bollinger et al., 2020). It seems that the degradation ability of *Pseudomonas* is partly due to the high hydrophobicity of the cell surface, which is conducive to its adhesion to the polymer, and the formation of biofilm to promote degradation. In addition, the secretion of extracellular polysaccharide is important to promote the degradation of LDPE. However, the degradation effects vary with types of plastics (Shah et al., 2008).

Biofilm has an important effect on biodegradation, which relies on the influence of nutritional conditions for its formation. An alteration of *Pseudomonas* surface hydrophobicity was detected by the change of glucose and ammonium sulfate concentrations (Tribedi and Sil, 2014). Thus, the increased cell surface hydrophobicity promoted the efficient formation of biofilm in a polyethylene succinate film, which was positively correlated to degradation (Tribedi and Sil, 2014). Conversely, in the presence of marine sediments, which were organic carbon-rich, biofilm formation was diminished, and the degradation of PE was minimal (Nauendorf et al., 2016). Therefore, environmental and nutritional conditions that favor the genesis of biofilms on plastic polymers are important stimuli for the degradation of synthetic plastics by *Pseudomonas* (Wilkes and Aristilde, 2017).

A toxic metabolite produced by one microorganism may be a substrate for the growth of another, thus eliminating the toxic effects to the former (Yuan et al., 2020). Conversely, symbiotic interactions in consortia increased the activity and tolerance for the treatment of pollutants (Singh and Wahid, 2015; Yuan et al., 2020). For example, *Bacillus* and *Paenibacillus* reduced the dry weight of MP particles (14.7% after 60 d) and the mean particle diameter (22.8% after 60 d) when in a bacterial consortium (Park and Kim, 2019). Moreover, a marine bacterial community composed of *Idiomarina*, *Marinobacter*, *Exiguobacterium*, *Halomonas*, and *Ochrobactrum* was capable of colonizing and degrading PET and PE efficiently (Gao and Sun, 2021).

### 3.2. Mechanism and enzymes of microplastic biodegradation

**Figure 3:**
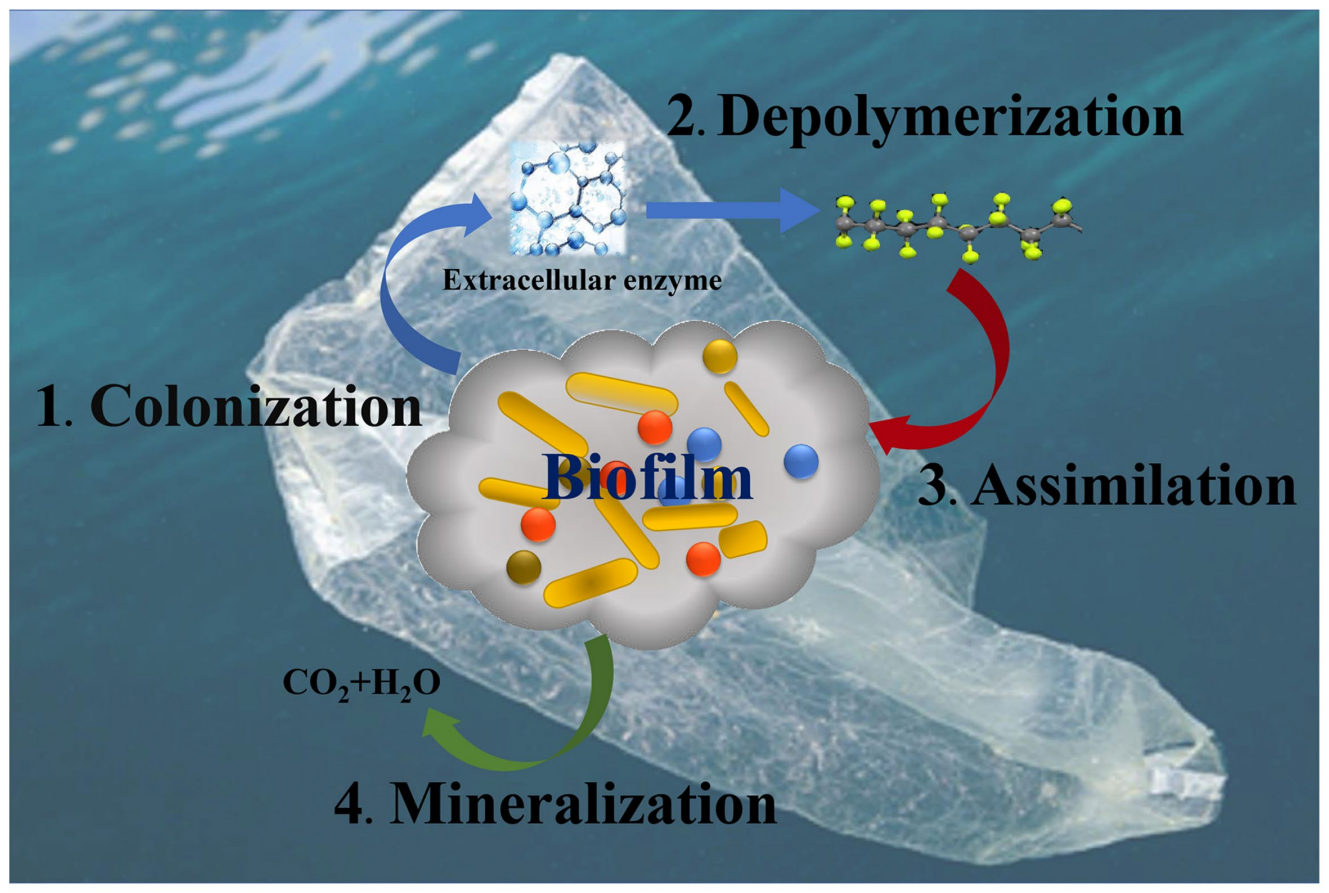
The mechanism of microplastic biodegradation.

The mechanisms and enzymes of MP biodegradation have not been fully elucidated. Overall, the process of microbial degradation of MPs may be divided into colonization, depolymerization, assimilation and mineralization (Pathak, 2017), which involve multiple biochemical reactions. After colonization on the surface of MPs, microorganisms release extracellular enzymes to combine with polymers and cleave them. This leads to depolymerization into low molecular weight oligomers, dimers and even monomers (Shah et al., 2008). The processes involve hydrolysis (for hydrolyzable plastics) and oxidative degradation (for hydrolyzable and non hydrolyzable plastics). After depolymerization, the oligomers enter the cells, and serve as the carbon source for microbial growth, which is assimilation. The mineralization refers to the decomposition of different types of MPs into small molecules, such as CO_2_, CH_4_ and H_2_O, through the TCA cycle (Figure 3). Up to now, the plastic degrading enzymes have been reported to include hydrolases (such as esterase, lipase, keratinase, and cutinase) and oxidoreductases (such as laccase, manganese peroxidase, hydroxylase and lignin peroxidases) (Gan and Zhang, 2019; Zhang et al., 2021a).

PE degrading biocatalysts include hydroxylase, laccase, peroxidase and reductase (Amobonye et al., 2021). A manganese peroxidase was identified in two types of fungi leading to degradation of PE (Iiyoshi et al., 1998). Laccase, which is produced by *Actinomycetes*, *Rhodococcus*, *Aspergillus flavus* and *Pleurotus ostreatus,* degraded PE significantly (Santo et al., 2013; Gómez-Méndez et al., 2018; Zhang et al., 2020). With *Pseudomonas aeruginosa*, alkane hydroxylase was involved directly in the biodegradation of LMWPE. Also, rubredoxin and rubredoxin reductase exerted an indirect role through the transfer of related electrons (Jeon and Kim, 2015). In addition, alkane hydroxylase, which was produced by *Pseudomonas* sp. E4, played an important role in the degradation of PE through the oxidation of the PE main chain (Yoon et al., 2012).

Although the microbial degradation of PS by various bacteria and fungi has been demonstrated, the major enzymes involved in the initial depolymerization have not been clearly identified (Amobonye et al., 2021). An extracellular esterase from *Lentinus tigrinus* (Tahir et al., 2013) and certain polymerases from *Bacillus* and *Pseudomonas* (Mohan et al., 2016) have been shown to degrade PS. It was determined that styrene monooxygenases (SMO), styrene oxide isomerase (SOI), phenylacetaldehyde dehydrogenase (PAD) and multiple enzymes of phenylacetate degradation (PAA#) could degrade PS into styrene oxide, phenylacetaldehyde, phenylacetate and phenylacetic acid (an intermediate product of TCA cycle; Tischler et al., 2009).

Many hydrolases, including lipase, esterase and keratinase, showed PET degrading ability (Jabloune et al., 2020). *Ideonella sakaiensis* produced two enzymes, PETase and MHETase (encoded by *ISF6_4831* and *ISF6_0224*), hydrolyzing PET efficiently into two environmentally benign monomers, i.e., terephthalic acid (TPA) and ethylene glycol (EG; Yoshida et al., 2016). PETase contained a Ser-His-Asp catalytic triad at the active site, and its catalytic mechanism was revealed (Joo et al., 2018). Then, PETase was found have structural characteristics of lipase and cutinase, and a method for degradation of high crystallinity PET catalyzed by this enzyme was established (Austin et al., 2018). IsPETase variants with improved thermal stability and much higher PET degradation activities were developed (Son et al., 2020). In addition, a suberinase with remarkable stability and capability to hydrolyse PET to terephthalic acid and other polymers was sourced from *Streptomyces scabies* (Jabloune et al., 2020). Furthermore, a dual enzyme system composed of polyester hydrolase and carboxylesterase enhanced the biocatalytic degradation of PET films (Barth et al., 2016). Moreover, 504 possible PET hydrolase candidate genes from databases were identified, and 349 putative PET hydrolases were detected by analyzing 108 marine and 25 terrestrial metagenomes. PET hydrolase genes were found mainly in the phylum Bacteroidetes in marine metagenomes, and in Actinobacteria and Proteobacteria in terrestrial metagenomes (Danso et al., 2018). Four novel PET hydrolase genes were cloned, expressed heterologously and their biochemical traits were described, moreover, two displayed thermal stability and potential application value (Danso et al., 2018).

## 4. Plastisphere and quorum sensing

Quorum sensing (QS) refers to the cell-to-cell communication process that is mediated by the production, release, accumulation and group-wide detection of extracellular signaling molecules. These regulate biological behaviors, such as bioluminescence, secondary metabolite production and biofilm formation (Mukherjee and Bassler, 2019). Gram negative bacteria mainly rely on *N*-acylhomoserine lactones (AHLs) as their autoinducers (Williams et al., 2007), whereas Gram positive bacteria mostly use autoinduced peptides (Schaefer et al., 2008).

QS plays an important role in the formation of bacterial biofilms. QS will be induced when planktonic microorganisms proliferate to a certain cell density on a medium, promoting more bacteria to attach, leading to a mature biofilm (Costerton et al., 1995). It was demonstrated that the stages of bacterial biofilm formation, attachment, maturation, aggregation and dispersal, are always regulated by QS, which affects the structure of biofilms (Parsek and Greenberg, 2005). For example, *rhl*I/R and *las*I/R are AHL-dependent QS systems in *Pseudomonas aeruginosa*. *Rhl* system influences greatly the formation of biofilm (Mattmann et al., 2008). The *las* system determines the structural differences of bacterial biofilms, and exerts an important role in the irreversible adhesion phase of the film-forming process (Lin et al., 2012). Studies on *Burkholderia cepacia* (Brackman et al., 2009), *Aeromonas hydrophila* (Lynch et al., 2002), *Yersinia pseudotuberculosis* (Atkinson et al., 1999), *Vibrio cholerae* (Ye and Lu, 2010) and *Staphylococcus* (Kiran et al., 2008) showed that QS could regulate bacterial aggregation and surface adhesion. Furthermore, QS was found to play an important role in the degradation of marine organic particles by *Pantoea ananatis* and *Ruegeria mobilis* (Su et al., 2018). Also, quorum sensing is involved in marine snow, and its possible influence on production of extracellular hydrolytic enzymes in *Pantoea ananatis* B9 (Jatt et al., 2015).

**Figure 4:**
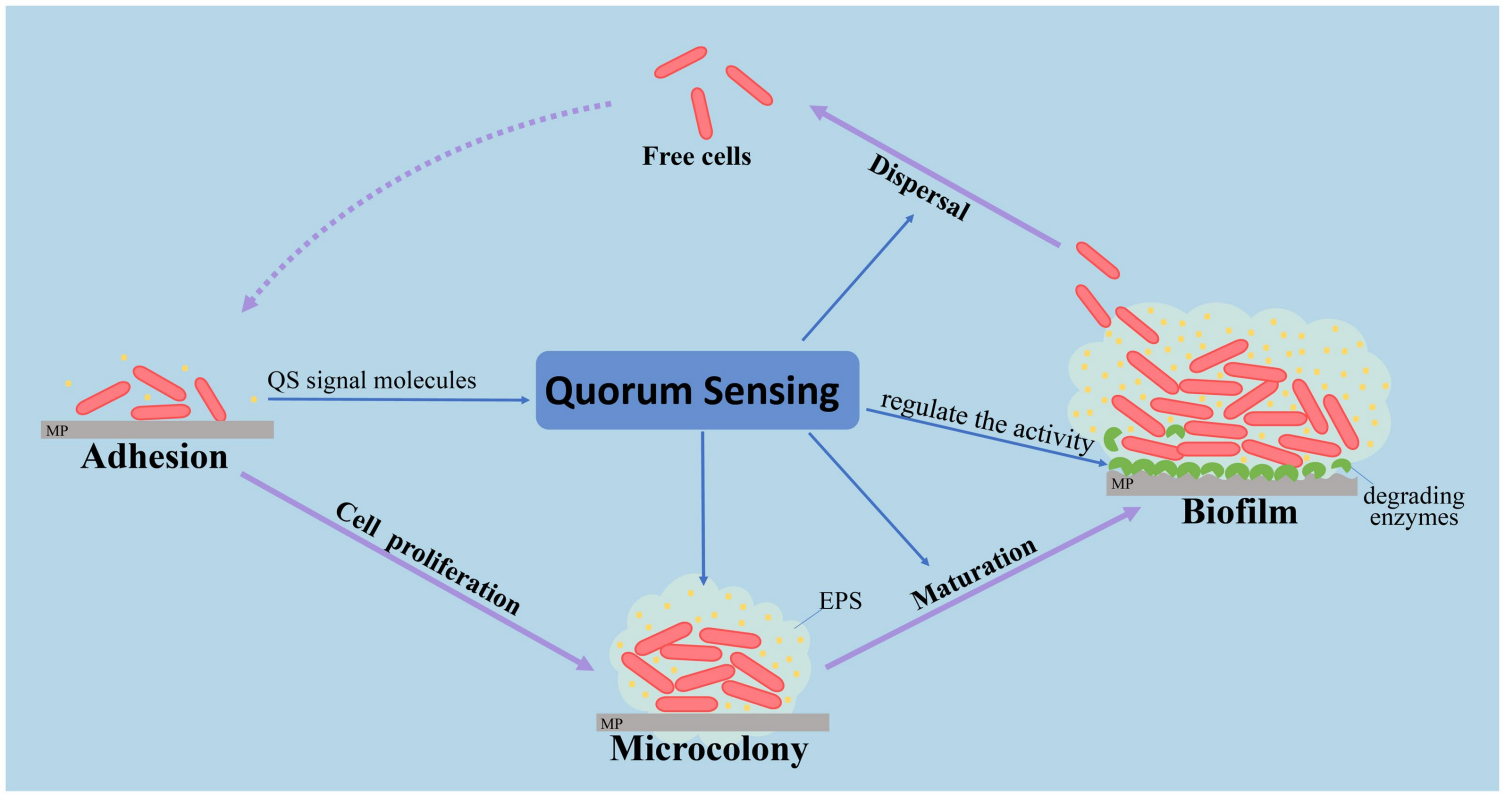
The regulation of quorum sensing in the plastisphere.

Although the relevant research has not yet occurred, it is reasonable to believe that QS has the potential to affect the formation and composition of the plastisphere. Studies have showed that *Rhodobacteraceae* occupied core populations in the plastisphere (Debroas et al., 2017; Dussud et al., 2018) and accounted for 16.4% in bacterial biofilms of MPs in the Yangtze River Estuary, China (Jiang et al., 2018). A high proportion of *Rhodobacteraceae* in the plastisphere was attributed to the conserved and widespread QS signaling molecules participating in various metabolic regulations, especially flagellum movement and biofilm formation on plastic particles (Zan et al., 2014). QS may help *Rhodobacteraceae* attach and aggregate on the surface of MPs, and inhibit other bacteria, including *Gammaproteobacteria*. The primary model of QS regulation to the plastisphere is shown in Figure 4. In addition, QS bacteria, which were isolated from MPs, exhibited strong biofilm-forming ability and favorable conditions for epiphytic growth. Thus, *Oceanicola* sp. strain D3 with QS ability was isolated from a PVC biofilm (Li et al., 2019). Interestingly, bacteriostasis, algae inhibition and the dimethylsulphoniopropionate (DMSP) degradation function of QS bacteria may impact the pollution of MPs in the marine environment (Gagné, 2017).

QS has the potential to be a new way to study marine MP biofilms and their degradation. On the one hand, QS has a profound impact on the microbial communities and the succession of biofilms, and the bacteria may have an important role in regulating the process of plastisphere formation. This may not only increase QS bacterial colonization on MPs by producing extracellular polysaccharides, but also inhibit the adhesion of other bacteria by producing antibacterial materials. Conversely, QS regulates many metabolic functions of dominant bacteria and enlarges their ecological function. For example, AHLs may change the activity of extracellular hydrolases (Jatt et al., 2015). Thus, a QS system based on AHLs may have a key role in the degradation of MPs. Moreover, bacterial interaction based on the QS network may provide a new perspective for the degradation of marine MPs. The dominant colonization and rapid sensitivity to organic substances may allow QS bacteria to be effective indicators for monitoring MP pollution in the marine environment. Consequently, understanding the plastisphere and promoting MPs degradation by QS is expected to become a new research perspective.

## 5. Future directives

Plastic pollution is a problem that needs to be solved urgently, and has gained growing public and scientific attention in recent years. In this review, the formation process and microbial community composition of plastisphere – the biofilm colonizing on plastic, the bacteria and the enzymes as well as their degrading mechanism of MPs are discussed. In particular, we clarified the potential role of QS in plastic research for the first time. This may well offer novel ideas to the study of plastic pollution control.

Microorganisms provide a new perspective for the exploration of plastic treatment, however, research on the plastisphere still needs to be advanced. The understanding of the distribution of MPs, the microbial community structure and formation process of the plastisphere and potential degrading bacteria of MPs need to be fully explored. Future studies could be categorized mainly into three themes. Firstly, more geographical distributions of MPs and biofilms and comparisons of different materials, types of plastics and plastic area/color are needed. These studies will help form the global distribution pattern of MPs, and composition of biofilms (similarities and differences), as well as promote regional targeted MP pollution control. Studying the heterogeneity of microbial community structure in the plastisphere and biofilms will lay the foundation for exploring the metabolism and MPs degradation ability of bacteria. Secondly, more investigations are needed on the effects of single bacterial strains and communities on degradation. Methods of exploring the characteristics of materials as the basal point, optimizing degradation conditions, using bioinformatics to analyze the environmental response of relevant metabolic genes in the habitat, as well as creating high-efficiency MP degrading bacterial consortia should be undertaken. Thirdly, new molecular techniques and *in-situ* characterization of isolated strains are needed. Specific degradation pathways and degradation enzymes need to be found to fully develop the known non-specific pathways to metabolize plastics. In addition, the study of QS could focus on the distribution, proportion and metabolism of QS bacteria in the plastisphere, which will help deepen the understanding of plastisphere and potential degrading bacteria of MP.

## Author contributions

MY and X-HZ: conceptualization. XZ: writing—original draft. XZ, MY, and X-HZ: writing—review and editing. X-HZ: resources and funding acquisition. All authors contributed to the article and approved the submitted version.

## Funding

This work was funded by the Scientific and Technological Innovation Project of Laoshan Laboratory (nos. LSKJ202203200 and 2022QNLM030004-3), the National Natural Science Foundation of China (nos. 41730530 and 92251303), and the Fundamental Research Funds for the Central Universities (no. 202172002).

## Conflict of interest

The authors declare that the research was conducted in the absence of any commercial or financial relationships that could be construed as a potential conflict of interest.

## Publisher’s note

All claims expressed in this article are solely those of the authors and do not necessarily represent those of their affiliated organizations, or those of the publisher, the editors and the reviewers. Any product that may be evaluated in this article, or claim that may be made by its manufacturer, is not guaranteed or endorsed by the publisher.
